# Supplementary material for: Interventions for depression and anxiety among people with diabetes mellitus: Review of systematic reviews
Source: PLoS One. 2023 Feb 9;18(2):e0281376. doi: 10.1371/journal.pone.0281376 (PMC9910656; doi:10.1371/journal.pone.0281376)
Supplement: S1 Table — (DOCX) [file pone.0281376.s001.docx]

**S1 Table. Characteristics of systematic reviews included (n=13).**

| **Systematic review**  **Country of study** | **N. of studies** | **N. of people** | **Age (years)**  **(range or mean ± SD)** | ***Psychological disorder** | **Intervention** | **Search period** | **Objectives of study** | |
| --- | --- | --- | --- | --- | --- | --- | --- | --- |
| **TYPE 2 DIABETES** | | | | | | | |  |
| Berhe et al., 2020 [24]  Ethiopia | 7 | 1,983 | Adults  (18–87) | Depression and depressive symptoms | Psychological | Jan/2009 to Jun/2020 | To analyze the literature for evidence of the effect of motivational interviewing intervention has on HbA1c and depression in adults with Type 2 diabetes | |
| Xie et al., 2017 [22]  China | 31 | 2,616 | Adults  (NR) | Depression and anxiety | Psychological/ Psychosocial | Mar/2000 to Mar/2017 | To explore the efficacy of psychosocial intervention in management of adults with Type 2 diabetes | |
| Perrin et al., 2019 [31]  United Kingdom | 32 | 5,213 | Adults  (40–70) | Depression and distress | Psychoeducational | 1946 to 2016 | To determine interventions that successfully address depression and/or diabetes-specific emotional distress and HbA1c in adults with Type 2 diabetes | |
| Hadjiconstantinou et al., 2016 [33]  United Kingdom | 9 | 3,612 | Adults  (24-67) | Depression, anxiety and distress | Web-based health education | 1995 to 2016 | To critically appraise and quantify the evidence on the effect of web-based interventions to improve well-being in people with Type 2 diabetes | |
| **TYPE 1 AND TYPE 2 DIABETES** | | | | | | | |  |
| Baumeister et al., 2012 [23]  Germany | 19 | 1,592 | Adults  (45–71) | Depression | Psychological, pharmacological and health education | Up to Dec 2011 | To determine the effects of this interventions for depression in adults with diabetes | |
| **Li et al., 2017 [25]  China | 10 | 998 | Any age  (NR) | Depression and anxiety | Psychological | Up to May 2016 | To examine the efficacy of CBT for people with diabetes and depression and to identify which aspects can be improved through intervention in these population | |
| Ni et al., 2020 [29]  China | 7 | 741 | ≥ 16  years old  (18-69) | Depression | Psychological | Up to Dec 2019 | To determine the effectiveness of MBCT and MBSR on depression in people with diabetes | |
| **Uchendu et al., 2016 [26]  United Kingdom | 09 | 1,445 | Adults  (37 ± 11) | Depression and anxiety | Psychological | 1806 to 2014 | To establish the effectiveness of CBT on glycemic values and comorbid diabetes-related distress, depression, anxiety and quality of life in the short, medium and longer term, among adults with diabetes | |
| Vanderfeltz-Cornelis et al., 2020 [30]  United Kingdom | 32 | 3,543 | Adults  (NR) | Depression | Psychological, pharmacological, and psychoeducational | Up to Aug 2019 | To provide an estimate of the effect of interventions on comorbid major depressive disorder or subthreshold depression in adults with diabetes | |
| Wang et al., 2017 [27]  China | 5 | 834 | Adults  (NR) | Depression | Psychological | Up to  Oct 2016 | To evaluate the effect of CBT in improving the depression symptoms of adults with diabetes | |
| Yang et al., 2020 [28]  China | 23 | 2,705 | Adults  (NR) | Depression | Psychological | 2007 to  Apr 2019 | To provide an overview of the effectiveness of CBT for improving glycemic values, psychological, and physiological outcomes in adults with diabetes | |
| Huang et al., 2013 [32]  China | 8 | 2,203 | Any age  (NR) | Depression | Psychoeducational | 1806 to 2013 | To examine whether collaborative care can improve depression and diabetes outcomes in people with both diseases | |
| Li et al., 2020 [34]  China | 12 | 822 | Any age  (20-68) | Depression | Pharmacological | Up to May 2019 | To evaluate the efficacy and safety of *Gardenia fructus* antidepressant formula for depression in people with diabetes | |

CBT (cognitive behavioural therapy). MBCT (mindfulness-based cognitive therapy). MBSR (mindfulness-based stress reduction). N (number). SD (Standard Deviation). NR (not reported)

*The tools/scales used by clinical trials to measure outcomes (depression, anxiety, among others) are available in

Supporting. Data information file **.**

**Systematic reviews that reported duration of diabetes. These reviews included population that have at least 6 months of diagnosis of diabetes mellitus).
