# Supplementary material for: Interventions for depression and anxiety among people with diabetes mellitus: Review of systematic reviews
Source: PLoS One. 2023 Feb 9;18(2):e0281376. doi: 10.1371/journal.pone.0281376 (PMC9910656; doi:10.1371/journal.pone.0281376)
Supplement: S3 Table — (DOCX) [file pone.0281376.s003.docx]

**S3 Table. Results of psychological interventions (n= 9 reviews).**

| **References** | **Outcomes** | **N. of people** | **Meta-analysis results (95% CI)** | **Author's results** | **Publication bias and quality of evidence** |
| --- | --- | --- | --- | --- | --- |
| **PSYCHOLOGICAL INTERVENTIONS (n= 4 reviews)** | | | | | |
| Berhe et al., 2020  [24] | **Reduction in depressive symptoms**  (Motivational Interviewing vs control group) - session time of 30 minutes  follow up was NR | 68 | WMD= -1.58  (-5.05 to -0.19)  I^2^=48% | Motivational Interviewing was not superior to control group | **PB=** absent (Egger’s test)  **GRADE=** NR |
|  | **Reduction in depressive symptoms**  (Motivational Interviewing vs control group) - session time of 60 minutes  follow up was NR | 1,031 | WMD= -4.30  (-9.32 to -0.73)  I^2^=95% | Motivational Interviewing was not superior to control group | **PB=** absent (Egger’s test)  **GRADE=** NR |
|  | **Reduction in depressive symptoms**  (Motivational Interviewing vs control group)  3 months follow up | 129 | WMD= -4.45  (-10.58 to 1.69)  I^2^=96% | Motivational Interviewing was not superior to control group | **PB=** absent (Egger’s test)  **GRADE=** NR |
|  | **Reduction in depressive symptoms**  (Motivational Interviewing vs control group)  24 months follow up | 970 | WMD= -2.12  (-5.54 to 1.30)  I^2^=83% | Motivational Interviewing was not superior to control group | **PB=** absent (Egger’s test)  **GRADE=** NR |
|  | **Reduction in HbA1c values**  (Motivational Interviewing vs control) in adults  follow up was NR | 1,428 | WMD= -0.27  (-0.46 to -0.09)  I^2^=38% | Motivational Interviewing was superior to control group | **PB=** absent (Egger’s test)  **GRADE=** NR |
| Ni et al., 2020 [29] | **Reduced depression scores**  (MBSR or MBCT vs control group)  follow up was NR | 641 | SMD= -0.84  (-1.16 to -0.51)  I^2^=72% | MBSR or MBCT was superior to control group | Publication bias= NR  **GRADE=** NR |
|  | **Score of quality of life** (mental health)  (MBSR or MBCT vs control group)  follow up was NR | 443 | MD= 7.06  (5.09 to 9.03)  I^2^=0% | MBSR or MBCT was superior to control group | Publication bias= NR  **GRADE=** NR |
|  | **Score of quality of life** (physical health)  (MBSR or MBCT vs control group  follow up was NR | 443 | MD= 3.14  (-0.38 to 6.67)  I^2^=73% | MBSR or MBCT was superior to control group | **PB=** NR  **GRADE=** NR |
|  | **Reduction in HbA1c values**  (MBSR or MBCT vs control group)  follow up was NR | 578 | MD= -0.28  (-0.47 to -0.09)  I^2^=0% | MBSR or MBCT was superior to control group | **PB=** NR  **GRADE=** NR |
| Vanderfeltz-Cornelis et al., 2020 [30] | **Reduced depression scores**  (Psychological Interventions vs control group)  follow up was NR | NR | SMD= 0.56  (0.42 to 0.70)  I^2^=NR | Psychological Interventions was superior to control group | **PB=** Small effect (Begg funnel plot)  **GRADE=** moderate to high |
|  | **Reduction in HbA1c values**  (Psychological Interventions vs control group)  follow up was NR | NR | SMD= 0.61  (0.15 to 1.07)  I^2^=NR | Psychological Interventions was superior to control group | **PB=** Small effect (Begg funnel plot)  **GRADE=** moderate to high |
| *Xie et al., 2017 [22] | **Reduced depression symptoms**  (Psychosocial vs control group) until 16 months follow up | 2,476 | SMD= -1.50  (-1.83 to -1.18)  I^2^=92% | Psychological Interventions was superior to control group | **PB=** NR  **GRADE=** NR |
|  | **Reduced anxiety symptoms**  (Psychosocial vs control group) until 16 months follow up | 871 | SMD= -1.18  (-1.50 to -0.85)  I^2^=79% | Psychological Interventions was superior to control group | **PB=** NR  **GRADE=** NR |
|  | **Reduction in HbA1c values**  (Psychosocial vs control group) until 16 months follow up | 1,765 | SMD= -0.81  (-1.10 to -0.53)  I^2^=87% | Psychological Interventions was superior to control group | **PB=** NR  **GRADE=** NR |
| **COGNITIVE BEHAVIORAL THERAPY (n= 5 reviews)** | | | | |  |
| Baumeister et al., 2012 [23] | **Reduction in depression scores**  (CBT vs usual care)  until 6 months follow up | 41 | SMD= -1.10  (-1.75 to -0.45)  I²=66% | CBT was superior to usual care | **PB=** NR  **GRADE=** NR |
|  | **Depression remission rate**  (CBT vs usual care)  until 6 months follow up | 41 | OR= 4.67  (1.25 to 17.44)  I²=7% | CBT was superior to usual care | **PB=** NR  **GRADE=** low |
|  | **Reduction in depression scores**  (CBT vs usual care)  until 6 months follow up | 42 | MD= -1.40%  (-2.60 to -0.20)  I²=78% | CBT showed a significant reduction compared to usual care | **PB=** NR  **GRADE=** low |
|  | **Reduction in depression scores**  (Telephone-delivered CBT vs usual care)  until 1 month of follow up | 291 | SMD= -0.42  (-0.66 to -0.18)  I²=86% | Telephone-delivered CBT was superior to usual care | **PB=** NR  **GRADE=** NR |
|  | **Depression remission rate**  (Telephone-delivered CBT vs usual care)  until 1 month of follow up | 291 | OR= 1.83  (1.12 to 3.00)  I²=57% | Telephone-delivered CBT was superior to usual care | **PB=** NR  **GRADE=** moderate |
|  | **Reduction in depression scores**  (Web-based CBT vs waiting-list) until 6 months follow up | 255 | SMD= -0.29  (-0.41 to -0.17)  I²=66% | Web-based CBT was superior to control group | **PB=** NR  **GRADE=** NR |
|  | **Depression remission rate**  (Web-based CBT vs waiting-list) until at 6 months follow up | 255 | OR= 2.20  (1.28 to 3.77)  I²=7% | Web-based CBT was superior to control group | **PB=** NR  **GRADE=** low |
|  | **Reduction in HbA1c values**  (Web-based CBT vs waiting-list control group)  until 6 months follow up | 255 | MD= 0.4%  (0.1 to 0.7)  I²=78% | Web-based CBT was inferior to the waiting-list | **PB=** NR  **GRADE=** low |
| Li et al., 2017 [25] | **Reduced depression scores**  (CBT vs usual care)  until 6 months follow up | 381 | SMD= −0.86  (−1.41 to − 0.31)  I^2^=81% | CBT had improvement compared to control group | **PB=** NR  **GRADE=** NR |
|  | **Reduced depression scores**  (CBT vs usual care)  until 12 months follow up | 719 | SMD = −0.38  (−0.57 to −0.19)  I^2^=81% | CBT had improvement compared to control group | **PB=** NR  **GRADE=** NR |
|  | **Reduced anxiety scores**  (CBT vs usual care)  until 6 months follow up | 135 | SMD = −0.04  (−0.76 to 0.67)  I^2^=77% | CBT did not show difference compared to control group | **PB=** NR  **GRADE=** NR |
|  | **Reduced anxiety scores**  (CBT vs usual care)  until 12 months follow up | 115 | SMD = −0.49  (−0.88 to −0.10)  I^2^=9% | CBT did not show difference compared to control group | **PB=** NR  **GRADE=** NR |
|  | **Reduction in HbA1c values**  (CBT vs usual care)  until 6 months follow up | 303 | SMD = −0.30  (−0.71 to 0.10)  I^2^=74% | CBT did not show difference compared to control group | **PB=** NR  **GRADE=** NR |
|  | **Reduction in HbA1c values**  (CBT vs usual care)  until 12 months follow up | 705 | SMD = −0.19  (−0.47 to 0.09)  I^2^=74% | CBT did not show difference compared to control group | **PB=** NR  **GRADE=** NR |
|  | **Improvement in quality of life**  (CBT vs usual care)  until 12 months follow up | 653 | SMD = 0.29  (0.08 to 0.51)  I^2^=38% | CBT had improvement compared to control group | **PB=** NR  **GRADE=** NR |
| Uchendu  et al., 2016 [26] | **Reduced depression scores**  (CBT vs control group)  until 8 months follow up | 487 | SMD= -0.43  (-0.79 to -0.06)  I^2^=73% | CBT had improvement compared to control group | **PB=** NR  **GRADE=** NR |
|  | **Reduced depression scores**  (CBT vs control group)  until 12 months follow up | 662 | SMD= -0.26  (-0.41 to -0.10)  I^2^=44% | CBT had improvement compared to control group | **PB=** NR  **GRADE=** NR |
|  | **Reduced anxiety scores**  (CBT vs control group)  until 8 months follow up | 194 | SMD= -0.56  (-0.85 to -0.27)  I^2^=0% | CBT had improvement compared to control group | **PB=** NR  **GRADE=** NR |
|  | **Reduced depression scores**  (CBT vs control group)  until 12 months follow up | 74 | SMD= -0.33  (-0.79 to 0.13)  I^2=^NR | CBT did not have significant effect compared to control group | **PB=** NR  **GRADE=** NR |
|  | **Reduction in HbA1c values**  (CBT vs usual care)  until 8 months follow up | 459 | SMD= -0,36  (-0.55 to -0.18)  I^2^=44% | CBT had a significant effect compared to control group | **PB=** NR  **GRADE=** NR |
|  | **Reduction in HbA1c values**  (CBT vs usual care)  until t 12 months follow up | 644 | SMD= -0,11  (-0.26 to 0.05)  I^2^=0% | CBT did not have significant effect compared to control group | **PB=** NR  **GRADE=** NR |
| Wang et al., 2017 [27] | **Reduced depression symptoms**  (CBT vs usual care)  post-intervention | 466 | SMD= –0.43  (-0.73 to -0.12)  I^2^=58% | CBT had a significant effect compared to control group | **PB=** absent (Egger's test and funnel plot)  **GRADE=** NR |
|  | **Reduced depression symptoms**  (CBT vs usual care)  until 12 months follow up | 455 | SMD= -0.38  (-0.54 to -0.23)  I^2^=25% | CBT had a significant effect compared to control group | **PB=** absent (Egger's test and funnel plot)  **GRADE=** NR |
| Yang et al., 2020 [28] | **Reduced depression symptoms**  (CBT vs control group)  follow up was NR | NR | MD= -2.79  (-4.45 to -1.03)  I^2^=97% | CBT had a significant effect compared to control group | **PB=** minimal (Egger's test and funnel plot)  **GRADE=** NR |
|  | **Reduction in HbA1c values**  (CBT vs control group)  follow up was NR | NR | MD= -0.27  (-0.44 to -0.12)  I^2^=87% | CBT had a significant effect compared to control group | **PB=** absent (Egger's test and funnel plot)  **GRADE=** NR |

95% CI (95% confidence interval). CBT (cognitive behavioural therapy). GRADE (grading of recommendations assessment, development and evaluation). HbA1c (haemoglobin A1c). I^2^ (heterogeneity). MBCT (mindfulness-based cognitive therapy). MBSR (mindfulness-based stress reduction). MD (mean). NR (not reported). OR (odds ratio). PB (publication bias). RR (risk ratio). SMD (standardised mean differences). WMD (weighted mean differences).

*Psychological Interventions reported: interpersonal therapy, problem solving therapy, behavioural therapy and cognitive behavioural therap. Control groups: Not reported.
