# Supplementary material for: Interventions for depression and anxiety among people with diabetes mellitus: Review of systematic reviews
Source: PLoS One. 2023 Feb 9;18(2):e0281376. doi: 10.1371/journal.pone.0281376 (PMC9910656; doi:10.1371/journal.pone.0281376)
Supplement: S4 Table — (DOCX) [file pone.0281376.s004.docx]

**S4 Table. Results of psychoeducational interventions (n= 3 reviews) and health education (n= 2 reviews).**

| **References** | **Outcomes** | **N. of people** | **Meta-analysis results (95% CI)** | **Author's results** | **Publication bias and quality of evidence** |
| --- | --- | --- | --- | --- | --- |
| **PSYCHOEDUCATIONAL INTERVENTIONS (n=3)** | | | | |  |
| *Huang et al., 2013 [32] | **Depression treatment response rate**  (Collaborative care *vs* usual care)  follow up was NR | 1,096 | RR= 1.96  (1.38 to 2.78)  I^2^= 59% | Increased treatment response rate in the Collaborative care group was significant | **PB**= NR  **GRADE=** low |
|  | **Depression treatment response rate**  (Collaborative care *vs* usual care)  until 6 months follow up | 1,118 | RR= 1.64  (1.28 to 2.10)  I^2^= 54% | Beneficial effect significant of Collaborative care group | Publication bias= NR  **GRADE=** low |
|  | **Depression treatment response rate**  (Collaborative care vs usual care)  until 12 months follow up | 1,096 | RR= 1.33  (1.05 to 1.68)  I^2^= 59% | Indicated a 33% relative increase in treatment response rate in Collaborative care group | **PB**= NR  **GRADE=** low |
|  | **Depression remission rate**  (Collaborative care group *vs* usual care)  until 6 months follow up | 595 | RR= 1.33  (1.01 to 1.75)  I^2^= 0% | Significant increase in the response with Collaborative care | **PB=** NR  **GRADE=** low |
|  | **Depression remission rate**  (Collaborative care group *vs* usual care)  until 12 months follow up | 579 | RR= 1.20  (0.93 to 1.55)  I^2^= 0% | Non-significant effect of depression remission rate | **PB=** NR  **GRADE=** low |
|  | **Depression remission rate**  (Collaborative care *vs* usual care)  until 24 months follow up | 552 | RR= 1.15  (0.87 to 1.52)  I^2^= 0% | Non-significant effect of Collaborative care | **PB=** NR  **GRADE=** unclear |
|  | **Adherence to antidepressants**  (Collaborative care group *vs* usual care)  follow up was NR | 891 | RR= 1.79  (1.19 to 2.69)  I^2^= 84% | Statistically significant positive effect to antidepressant medication | **PB=** NR  **GRADE=** low |
|  | **Adherence to antidepressants and oral hypoglycaemic agents**  (Collaborative care group *vs* usual care)  follow up was NR | 238 | RR= 2.18  (1.61 to 2.96)  I^2^= 0% | Significant positive effect on rates of adherence to medication | **PB=** NR  **GRADE=** low |
|  | **Reduction in HbA1c values**  (Collaborative care group *vs* usual care)  until 6 months follow up | 1,101 | MD= -0.06  (-0.24 to 0.12)  I^2^= 16% | Non-significant reduction in HbA1c values in favour of Collaborative care | **PB=** NR  **GRADE=** low |
|  | **Reduction in HbA1c values**  (Collaborative care group *vs* usual care)  until 12 months follow up | 1,053 | MD= -0.07  (-0.28 to 0.13)  I^2^= 36% | Non-significant reduction in HbA1c values in favour of Collaborative care | **PB=** NR  **GRADE=** low |
| **Perrin et al., 2019 [31] | **Reduction of diabetes-specific emotional distress**  (Psychoeducational group *vs* control group)  follow up was NR | 5,206 | SMD= -0.13  (-0.25 to -0.01)  I^2^= 77% | Significant effect significant of psychoeducational interventions | **PB=** absent (Egger's test and funnel plot)  **GRADE=** low |
|  | **Reduction in HbA1c values** (Psychoeducational group *vs* control group) follow up was NR | 5,206 | SMD= -0.28  (-0.48 to -0.08)  I^2^= 71% | Significant effect significant of psychoeducational interventions | **PB=** absent (Egger's test and funnel plot)  **GRADE=** low |
| Vanderfeltz-Cornelis et al., 2020 [30] | **Reduced depression scores**  (Collaborative care vs control group)  follow up was NR | NR | SMD= 0.43  (0.28 to 0.58)  I^2^= NR | Collaborative care had a moderate effect compared to control group | **PB=** small effect (Begg funnel plot)  **GRADE=** moderate |
|  | **Reduction in HbA1c values**  (Collaborative care vs control group)  follow up was NR | NR | SMD= 0.21  (0.05 to 0.36)  I^2^= NR | Collaborative care had a small effect compared to control group | **PB=** small effect (Begg funnel plot)  **GRADE=** moderate |
| **HEALTH EDUCATION (n=2)** | | | | |  |
| Baumeister et al., 2012 [23] | **Depression remission rate**  (Health education vs usual care) until 12 months follow up | 59 | OR= 3.33  (1.14 to 9.75)  I²= 57% | Health education was superior to usual care | **PB=** NR  **GRADE=** moderate |
|  | **Reduction in HbA1c values**  (Health education vs usual care) until 12 months follow up | 59 | MD= -2.0  (-3.1 to -1.0)  I²= 83% | Health education was superior to usual care | **PB=** NR  **GRADE=** low |
| Hadjiconstantinou et al., 2016 [33] | **Reduced depression scores**  (Web-based interventions vs usual care)  until 18 months follow up | 1,321 | SMD= -0.31  (-0.73 to 0.11)  I^2^= 89% | Effect was not significant compared to control | **PB=** absent (Egger's test and funnel plot)  **GRADE=** NR |
|  | **Reduced distress scores**  (Web-based interventions vs usual care)  until 18 months follow up | 2,167 | SMD= -0.11  (-0.38 to 0.16)  I^2^= 88% | Effect was not significant compared to control | **PB=** absent (Egger's test and funnel plot)  **GRADE=** NR |

95% CI (95% confidence interval). GRADE (grading of recommendations assessment, development and evaluation). HbA1c (haemoglobin A1c). I^2^ (heterogeneity). MD (mean difference). NR (not reported). OR (odds ratio). PB (publication bias). RR (risk ratio). SMD (standardised mean difference).

*Interventions reported: multi-professional patient care; structured management plan; scheduled patient follow-up; enhanced inter-professional communication. Control groups reported: no additional intervention; usual care.

**Interventions reported: not reported. Control groups: not reported.
