# Supplementary material for: Interventions for depression and anxiety among people with diabetes mellitus: Review of systematic reviews
Source: PLoS One. 2023 Feb 9;18(2):e0281376. doi: 10.1371/journal.pone.0281376 (PMC9910656; doi:10.1371/journal.pone.0281376)
Supplement: S5 Table — (DOCX) [file pone.0281376.s005.docx]

**S5 Table. Results of pharmacological interventions (n= 3 reviews).**

| **References** | **Outcomes** | **N. of people** | **Meta-analysis results**  **(95% CI)** | **Author's results** | **Publication bias and quality of evidence** |
| --- | --- | --- | --- | --- | --- |
| Baumeister et al., 2012 [23] | **Improvement depression severity scores**  (Pharmacological interventions vs placebo)  until 6 months follow up | 306 | SMD=-0.61  (-0.94 to -0.27)  I^2^= 47% | Beneficial effect of antidepressants | **PB=** NR  **GRADE=** NR |
|  | **Improvement depression severity scores**  (SSRIs vs placebo)  until 6 months follow up | 241 | SMD= -0.39  (-0.64 to -0.13)  I^2^= 0% | Beneficial effect of SSRIs | **PB=** NR  **GRADE=** NR |
|  | **Depression remission rate** (Pharmacological interventions vs placebo)  until 6 months follow up | 136 | OR= 2.50  (1.21 to 5.15)  I^2^= 0% | Beneficial effect of antidepressants | **PB=** NR  **GRADE=** low |
|  | **Depression remission rate** (SSRIs vs placebo) - short-term | 108 | OR= 2.52  (1.11 to 5.75)  I^2^= 0% | Beneficial effect of SSRIs | **PB=** NR  **GRADE=** NR |
|  | **Reduction in HbA1c values** (Pharmacological interventions vs placebo)  until 6 months follow up | 238 | MD= -0.4%  (-0.6 to -0.1)  I^2^= 0% | Beneficial effect of antidepressants | **PB=** NR  **GRADE=** low |
| Li et al., 2020 [34] | **Improvement depression severity scores**  (*Gardenia Fructus* antidepressant formula vs no antidepressant)  follow up was NR | 71 | RR= -2.53  (-4.80 to -0.27)  I^2^= 96% | *Gardenia Fructus* antidepressant formula had lower HAMD scores compared to control group | **PB=** NR  **GRADE=** NR |
|  | **Improvement depression severity scores**  (*G. Fructus* antidepressant vs SSRIs)  follow up was NR | 225 | RR= -0.62  (-1.07 to -0.18)  I^2^= 80% | *Gardenia Fructus* antidepressant formula had lower HAMD scores compared to SSRI group | **PB=** NR  **GRADE=** NR |
|  | **Depression treatment response rate**  (*G. Fructus* antidepressant vs no antidepressant)  follow up was NR | 74 | RR= 1.66  (0.97 to 2.83)  I^2^= 80% | *Gardenia Fructus* antidepressant formula did not significantly increase response rate compared to control group | **PB=** NR  **GRADE=** NR |
|  | **Depression treatment response rate**  (*G. Fructus* antidepressant vs SSRIs)  follow up was NR | 79 | RR= 1.12  (0.96 to 1.31)  I^2^= 0% | *G. Fructus* antidepressant formula did not significantly increase response rate compared to SSRI | **PB=** NR  **GRADE=** NR |
| Vanderfeltz-Cornelis et al., 2020 [30] | **Reduced depression scores**  (Pharmacological vs control group)  follow up was NR | NR | ES= 0.57  (0.35 to 0.79)  I^2^= NR | Pharmacological had a significant effect compared to control group | **PB=** small effect (Begg funnel plot)  **GRADE=** moderate to high |
|  | **Reduction in HbA1c values**  (Pharmacological vs control group)  follow up was NR | NR | ES= 0.99  (0.13 to 1.80)  I^2^= NR | Pharmacological had a small effect compared to control group | **PB=** small effect (Begg funnel plot)  **GRADE=** moderate to high |

95% CI (95% confidence interval). I^2^ (heterogeneity). GRADE (Grading of Recommendations Assessment, Development and Evaluation). HAMD (Hamilton Depression Scale). HbA1c (haemoglobin A1c). MD (Mean Difference). NR (Not Reported). OR (odds ratio). PB (publication bias). RR (risk ratio). SMD (standardised mean difference). SSRI (Specific Serotonin Reuptake Inhibitors).
