# Supplementary material for: Interventions for depression and anxiety among people with diabetes mellitus: Review of systematic reviews
Source: PLoS One. 2023 Feb 9;18(2):e0281376. doi: 10.1371/journal.pone.0281376 (PMC9910656; doi:10.1371/journal.pone.0281376)
Supplement: S2 File — (DOCX) [file pone.0281376.s007.docx]

**Supporting**. Characteristics of excluded studies

| Study | Reason for exclusion |
| --- | --- |
| AZAMI G, SOH KL, SAZLINA SG, SALMIAH MS, AAZAMI S. Behavioral interventions to improve self-management in Iranian adults with type 2 diabetes: a systematic review and meta-analysis. J Diabetes Metab Disord 2018;17:365–80. | Other population |
| BASSI, G. et al. Assessment of Psychological Distress in Adults With Type 2 Diabetes Mellitus Through Technologies : Literature Review Corresponding Author : v. 23, 2021. | Other design of study |
| BASSI G, MANCINELLI E, DELL’ARCIPRETE G, RIZZI S, GABRIELLI S, SALCUNI S. Efficacy of eHealth Interventions for Adults with Diabetes: A Systematic Review and Meta-Analysis. Int J Environ Res Public Health. 2021;18(17). | Other population |
| BEATTY, L.; LAMBERT, S. A systematic review of internet-based self-help therapeutic interventions to improve distress and disease-control among adults with chronic health conditions. Clinical Psychology Review, v. 33, n. 4, p. 609–622, 2013. | Other design of study |
| BRIERLEY, S. et al. Psychological interventions for young people with Type 1 diabetes: A metaanalysis. Diabetic Medicine, v. 30, p. 177-178. 2013 | Not found full text |
| CELIK, A.; FORDE, R.; STURT, J. The impact of online self-management interventions on midlife adults with type 2 diabetes: A systematic review. British Journal of Nursing, v. 29, n. 5, p. 266–272, 2020. | Other outcomes |
| CEZARETTO, A. Interdisciplinariedade na resposta a intervenções em hábitos de vida para redução de risco cardiometabólico e a influência da depressão. p. 141, 2015. | Other design of study |
| CEZARETTO A, FERREIRA SRG, SHARMA S, SADEGHIRAD B, KOLAHDOOZ F. Impact of lifestyle interventions on depressive symptoms in individuals at-risk of, or with, type 2 diabetes mellitus: A systematic review and meta-analysis of randomized controlled trials. Nutr Metab Cardiovasc Dis 2016;26:649–62. | Other outcomes |
| CHAPMAN A, LIU S, MERKOURIS S, ENTICOTT JC, YANG H, BROWNING CJ, et al. Psychological interventions for the management of glycemic and psychological outcomes of type 2 diabetes mellitus in China: A systematic review and meta-analyses of randomized controlled trials. Front Public Heal 2015;3:1–22. | Other population |
| CHEN YY, TIAN Y, SUN XH, ZHANG FL, HUANG X. Effects of motivational interviewing on HbA1c and depression among cases with type 1 diabetes: a meta-analysis. Int J Diabetes Dev Ctries. Tianjin Vocat Inst, 2 Luohe Rd, Tianjin 300410, Peoples R China; 2022;42(2):191‑202. | Other population |
| CHEW, B. H. et al. Psychological interventions for diabetes-related distress in adults with type 2 diabetes mellitus. Cochrane Database of Systematic Reviews, v. 2015, n. 1, 2015. | Other population |
| EFTHYMIADIS A, BOURLAKI M, BASTOUNIS A. The effectiveness of psychological interventions on mental health and quality of life in people living with type 1 diabetes: a systematic review and meta-analysis. Diabetol Int. Japan; 2022;13(3):513‑21. | Other population |
| ELLIOTT, S. Cognitive behavioural therapy and glycaemic control in diabetes mellitus. Practical Diabetes, v. 29, n. 2, p. 67–71, 2012. | Other design of study |
| GARNER K, BOGGISS A, JEFFERIES C, SERLACHIUS A. Digital health interventions for improving mental health outcomes and wellbeing for youth with type 1 diabetes: A systematic review. Pediatr Diabetes. A. Serlachius, Department of Psychological Medicine, School of Medicine, University of Auckland, Auckland, New Zealand; 2022;23(2):258‑69. | Other population |
| GUTIERREZ, A. P. et al. Effectiveness of Diabetes Self-Management Education Programs for US Latinos at Improving Emotional Distress: A Systematic Review. Diabetes Educator, v. 45, n. 1, p. 13–33, 2019. | Other design of study |
| HARKNESS, E. et al. Identifying psychosocial interventions that improve both physical and mental health in patients with diabetes: A systematic review and meta-analysis. Diabetes Care, v. 33, n. 4, p. 926–930, 2010. | Other design of study |
| HONGASANDRA, N. R. et al. Effectiveness of yoga for patients with diabetes mellitus. Current Science, v. 113, n. 7, p. 1337–1353, 2017. | Other population |
| HUFFMAN, J. C. et al. Positive psychological interventions for patients with type 2 diabetes: Rationale, theoretical model, and intervention development. Journal of Diabetes Research, v. 2015, 2015. | Other design of study |
| ISMAIL, K.; WINKLEY, K.; RABE-HESKETH, S. Ismail2004_Lancet 363_1589-97.pdf. Lancet, v. 363, p. 1589–97, 2004. | Other outcomes |
| JENKINS, D. J. Psychological, physiological, and drug interventions for type 2 diabetes. The Lancet, v. 363, n. 9421, p. 1569–1570, maio 2004. | Other design of study |
| JENKINSON E, HACKETT RA, KNOOP I, HUDSON JL, MOSS- R. related distress : A systematic review and meta- ­ analysis. 2022;(April):1‑20. | Other population |
| JEREMIAH, O. J. et al. Evaluation of the effect of insulin sensitivity-enhancing lifestyle- A nd dietary-related adjuncts on antidepressant treatment response: Protocol for a systematic review and meta-analysis. Systematic Reviews, v. 8, n. 1, p. 1–11, 2019. | Other design of study |
| JESSE, C. D.; CREEDY, D. K.; ANDERSON, D. J. Effectiveness of psychological interventions for women with type 2 diabetes who are overweight or obese: A systematic review protocol. JBI Database of Systematic Reviews and Implementation Reports, v. 17, n. 3, p. 281–289, 2019. | Other design of study |
| JEWELL, R. R.; GOREY, K. M. Psychosocial interventions for emergent adults with type 1 diabetes: Near-empty systematic review and exploratory meta-analysis. Diabetes Spectrum, v. 32, n. 3, p. 249–256, 2019. | Other outcomes |
| KOK, J. L. A.; WILLIAMS, A.; ZHAO, L. Psychosocial interventions for people with diabetes and co-morbid depression. A systematic review. International Journal of Nursing Studies, v. 52, n. 10, p. 1625–1639, 2015. | Other design of study |
| KONG LN, HU P, ZHAO QH, YAO HY, CHEN SZ. Effect of peer support intervention on diabetes distress in people with type 2 diabetes: A systematic review and meta-analysis. Int J Nurs Pract. v. 26, n. 5, e12830, 2020. | Other population |
| LEE, S. W. H. et al. Interventions for people with type 2 diabetes mellitus fasting during Ramadan. Cochrane Database of Systematic Reviews, v. 2018, n. 11, 2018. | Other design of study |
| LEE HJ, LEE M, HA JH, LEE Y, YUN J. Effects of healthcare interventions on psychosocial factors of patients with multimorbidity: A systematic review and meta-analysis. Arch Gerontol Geriatr. v. 25, n. 91, 104241, 2020 . | Other population |
| LOPRESTI, A. L. Cognitive behaviour therapy and inflammation: A systematic review of its relationship and the potential implications for the treatment of depression. Australian and New Zealand Journal of Psychiatry, v. 51, n. 6, p. 565–582, 2017. | Other design of study |
| LIU F, GUAN Y, LI X, XIE Y, HE J, ZHOU ZG, et al. Different Effects of Structured Education on Glycemic Control and Psychological Outcomes in Adolescent and Adult Patients with Type 1 Diabetes: A Systematic Review and Meta-Analysis. Int J Endocrinol 2020;2020. | Other population |
| MARKOWITZ, S. M. et al. A review of treating depression in diabetes: Emerging findings. Psychosomatics, v. 52, n. 1, p. 1, 2011. | Other design of study |
| MARTINEZ, K. et al. Psychological factors associated with diabetes self-management among adolescents with Type 1 diabetes: A systematic review. Journal of Health Psychology, v. 23, n. 13, p. 1749–1765, 2018. | Other design of study |
| MATHIESEN, A. S. et al. Psychosocial interventions for reducing diabetes distress in vulnerable people with type 2 diabetes mellitus: A systematic review and meta-analysis. Diabetes, Metabolic Syndrome and Obesity: Targets and Therapy, v. 12, p. 19–33, 2019. | Other outcomes |
| MCBAIN, H. et al. Self-management interventions for type 2 diabetes in adult people with severe mental illness. Cochrane Database of Systematic Reviews, v. 2014, n. 11, 2014. | Other population |
| MCCOY, M. A.; THEEKE, L. A. A systematic review of the relationships among psychosocial factors and coping in adults with type 2 diabetes mellitus. International Journal of Nursing Sciences, v. 6, n. 4, p. 468–477, 2019. | Other design of study |
| MUHAMMAD A, LILIANTY E, MASYITHA A. Diabetes & Metabolic Syndrome : Clinical Research & Reviews Cognitive Behavioral Therapy for self-care behaviors with type 2 diabetes mellitus patients : A systematic review. Diabetes Metab Syndr Clin Res Rev. Diabetes India; 2022;16(7):102538. | Other population |
| MUSSELMAN, D. L. et al. Relationship of depression to diabetes types 1 and 2: Epidemiology, biology, and treatment. Biological Psychiatry, v. 54, n. 3, p. 317–329, 2003. | Other design of study |
| NASKAR, S.; VICTOR, R.; NATH, K. Depression in diabetes mellitus—A comprehensive systematic review of literature from an Indian perspective. Asian Journal of Psychiatry, v. 27, p. 85–100, 2017. | Other outcomes |
| NOORDALI, F.; CUMMING, J.; THOMPSON, J. L. Effectiveness of mindfulness-based interventions on physiological and psychological complications in adults with diabetes: A systematic review. Journal of Health Psychology, v. 22, n. 8, p. 965–983, 2017. | Other design of study |
| O’HARA, M. C. et al. A systematic review of interventions to improve outcomes for young adults with Type 1 diabetes. Diabetic Medicine, v. 34, n. 6, p. 753–769, 2017. | Other outcomes |
| PADILLA, V. L. et al. Neurocognitive impairment in patients with comorbid diabetes mellitus and depression. Personalized Medicine in Psychiatry, v. 1–2, p. 2–10, 2017. | Other design of study |
| POUWER F, SCHRAM MT, IVERSEN MM, NOUWEN A, HOLT RIG. How 25 years of psychosocial research has contributed to a better understanding of the links between depression and diabetes. Diabet Med. v. 37, n. 3, p. 383-392, 2020. | Other design of study |
| RAYNER, L. et al. Antidepressants for depression in physically ill people. Cochrane Database of Systematic Reviews, n. 4, 2008. | Other population |
| RESURRECCI DM, NAVAS-CAMPAÑA D, GUTI R. Psychotherapeutic Interventions to Improve Psychological Adjustment in Type 1 Diabetes : A Systematic Review. 2021 | Other population |
| REY VELASCO, E. et al. Pre-empting the challenges faced in adolescence: A systematic literature review of effects of psychosocial interventions for preteens with type 1 diabetes. Endocrinology, Diabetes and Metabolism, v. 3, n. 2, 2020. | Other outcomes |
| SCHMIDT, C. B. et al. Systematic review and meta-analysis of psychological interventions in people with diabetes and elevated diabetes-distress. Diabetic Medicine, v. 35, n. 9, p. 1157–1172, 2018. | Other population |
| STEED, L.; COOKE, D.; NEWMAN, S. A systematic review of psychosocial outcomes following education, self-management and psychological interventions in diabetes mellitus. Patient Education and Counseling, v. 51, n. 1, p. 5–15, 2003. | Other design of study |
| SUMLIN, L. L. et al. Depression and Adherence to Lifestyle Changes in Type 2 Diabetes: A Systematic Review. The Diabetes Educator, v. 40, n. 6, p. 731–744, 2014. | Other outcomes |
| THABREW, H. et al. E-Health interventions for anxiety and depression in children and adolescents with long-term physical conditions. Cochrane Database of Systematic Reviews, v. 2018, n. 8, 2018. | Other population |
| TOUMPANAKIS, A.; TURNBULL, T.; ALBA-BARBA, I. Effectiveness of plant-based diets in promoting well-being in the management of type 2 diabetes: A systematic review. BMJ Open Diabetes Research and Care, v. 6, n. 1, 2018. | Other design of study |
| UDEDI, M. et al. The effect of depression management on diabetes and hypertension outcomes in low- and middle-income countries: A systematic review protocol 11 Medical and Health Sciences 1117 Public Health and Health Services 11 Medical and Health Sciences 1103 Clinical . Systematic Reviews, v. 7, n. 1, p. 1–5, 2018. | Other design of study |
| VARELA-MORENO, E. et al. Effectiveness of eHealth-Based Psychological Interventions for Depression Treatment in Patients With Type 1 or Type 2 Diabetes Mellitus: A Systematic Review. FRONTIERS IN PSYCHOLOGY, v. 12, 2022. | Other design of study |
| VAN DER FELTZ-CORNELIS, C. M. et al. Effect of interventions for major depressive disorder and significant depressive symptoms in patients with diabetes mellitus: A systematic review and meta-analysis. General Hospital Psychiatry, v. 32, n. 4, p. 380–395, 2010. | Primary studies described in review with most current publication date |
| VAN DER HEIJDEN, M. M. P. et al. Effects of exercise training on quality of life, symptoms of depression, symptoms of anxiety and emotional well-being in type 2 diabetes mellitus: A systematic review. Diabetologia, v. 56, n. 6, p. 1210–1225, 2013. | Other design of study |
| VAN LAAKE-GEELEN, C. C. M. et al. The effect of exercise therapy combined with psychological therapy on physical activity and quality of life in patients with painful diabetic neuropathy: A systematic review. Scandinavian Journal of Pain, v. 19, n. 3, p. 433–439, 2019. | Other population |
| WANG, F. et al. The effects of qigong on anxiety, depression, and psychological well-being: A systematic review and meta-analysis. Evidence-based Complementary and Alternative Medicine, v. 2013, 2013. | Other outcomes |
| WANG, M. Y. et al. A systematic review of the efficacy of non-pharmacological treatments for depression on glycaemic control in type 2 diabetics. Journal of Clinical Nursing, v. 17, n. 19, p. 2524–2530, 2008. | Other design of study |
| WINKLEY K, LANDAU S, EISLER I, ISMAIL K. Psychological interventions to improve glycaemic control in patients with type 1 diabetes: Systematic review and meta-analysis of randomised controlled trials. Br Med J 2006;333:65–8. | Other population |
| WINKLEY K, UPSHER R, STAHL D, POLLARD D, BRENNAN A, HELLER S, et al. Systematic review and meta-analysis of randomized controlled trials of psychological interventions to improve glycaemic control in children and adults with type 1 diabetes. Diabet Med 2020;37:735–46. | Other population |
| YU, X.; CHAU, J. P. C.; HUO, L. The effectiveness of traditional Chinese medicine-based lifestyle interventions on biomedical, psychosocial, and behavioral outcomes in individuals with type 2 diabetes: A systematic review with meta-analysis. International Journal of Nursing Studies, v. 80, n. July 2017, p. 165–180, 2018. | Other population |
| ZHOU X, FANG M. E ects of traditional Chinese for type diabetes on psychological well-being : A systematic review and. | Other population |
| ZHOU, Z. et al. Effects of tai Chi on physiology, balance and quality of life in patients with type 2 diabetes: A systematic review and meta-analysis. Journal of Rehabilitation Medicine, v. 51, n. 6, p. 405–417, 2019. | Other outcomes |
