# Supplementary material for: Interventions for depression and anxiety among people with diabetes mellitus: Review of systematic reviews
Source: PLoS One. 2023 Feb 9;18(2):e0281376. doi: 10.1371/journal.pone.0281376 (PMC9910656; doi:10.1371/journal.pone.0281376)
Supplement: S3 File — (DOCX) [file pone.0281376.s008.docx]

**Supporting.** Flow diagram

**Identification of studies via databases and registers**

Records identified from databases (1,346):

MEDLINE (PubMed) (n = 226)

COCHRANE (n = 53)

WEB OF SCIENCE (n = 381)

EMBASE (n = 681)

LILACS (n = 5)

Other resources (n= 0)

Records removed *before screening*:

Duplicate records removed (n = 478)

**Identification**

Records screened (n = 868)

Reports not retrieved (n = 793)

Reports sought for retrieval

(n = 868)

**Screening**

Reports excluded (n= 62):

Not found full text (n =1)

Other population of study (n = 22)

Other outcomes (n = 13)

Other design of study (n = 25)

Primary studies described in review with most current publication date (n = 1)

Reports assessed for eligibility

(n = 75)

Systematic reviews included in overview

(n = 13)

**Included**

*From:*  Page MJ, McKenzie JE, Bossuyt PM, Boutron I, Hoffmann TC, Mulrow CD, et al. The PRISMA 2020 statement: an updated guideline for reporting systematic reviews. BMJ 2021;372:n71. doi: 10.1136/bmj.n71
