# Supplementary material for: Interventions for depression and anxiety among people with diabetes mellitus: Review of systematic reviews
Source: PLoS One. 2023 Feb 9;18(2):e0281376. doi: 10.1371/journal.pone.0281376 (PMC9910656; doi:10.1371/journal.pone.0281376)
Supplement: S4 File — (DOCX) [file pone.0281376.s009.docx]

**Supporting.** Search strategy

**Mesh terms**

**Diabetes Mellitus, Type 1**

(Autoimmune Diabetes) OR (Brittle Diabetes Mellitus) OR (IDDM) OR (Insulin Dependent Diabetes Mellitus) OR (Insulin Dependent Diabetes Mellitus 1) OR (Insulin-Dependent Diabetes Mellitus) OR (Insulin-Dependent Diabetes Mellitus 1) OR (Juvenile Onset Diabetes) OR (Juvenile Onset Diabetes Mellitus) OR (Juvenile-Onset Diabetes) OR (Juvenile-Onset Diabetes Mellitus) OR (Ketosis Prone Diabetes Mellitus) OR (Ketosis-Prone Diabetes Mellitus) OR (Sudden Onset Diabetes Mellitus) OR (Sudden-Onset Diabetes Mellitus) OR (Type 1 Diabetes) OR (Type 1 Diabetes Mellitus)

**Diabetes Mellitus, Type 2**

(Adult Onset Diabetes Mellitus) OR (Adult-Onset Diabetes Mellitus) OR (Ketosis Resistant Diabetes Mellitus) OR (Ketosis-Resistant Diabetes Mellitus) OR (Maturity Onset Diabetes) OR (Maturity Onset Diabetes Mellitus) OR (Maturity-Onset Diabetes) OR (Maturity-Onset Diabetes Mellitus) OR (MODY) OR (NIDDM) OR (Non Insulin Dependent Diabetes Mellitus) OR (Noninsulin Dependent Diabetes Mellitus) OR (Noninsulin-Dependent Diabetes Mellitus) OR (Non-Insulin-Dependent Diabetes Mellitus) OR (Slow Onset Diabetes Mellitus) OR (Slow-Onset Diabetes Mellitus) OR (Stable Diabetes Mellitus) OR (Type 2 Diabetes) OR (Type 2 Diabetes Mellitus) OR (Type II Diabetes Mellitus)

**Depression**

(Depression) OR (Depressions) OR (Depressive Symptom) OR (Depressive Symptoms) OR (Emotional Depression) OR (Emotional Depressions)

**Depressive Disorder**

(Depressive Disorder) OR (Depressive Disorders) OR (Depressive Neuroses) OR (Depressive Neurosis) OR (Depressive Syndrome) OR (Depressive Syndromes) OR (Endogenous Depression) OR (Endogenous Depressions) OR (Melancholia) OR (Melancholias) OR (Neurotic Depression) OR (Neurotic Depressions) OR (Unipolar Depression) OR (Unipolar Depressions)

**Anxiety**

(Anxiety) OR (Hypervigilance) OR (Nervousness) OR (Social Anxieties) OR (Social Anxieties) OR (Social Anxiety)

**Anxiety Disorders**

(Anxiety Disorder) OR (Anxiety Disorders) OR (Neurotic Anxiety State) OR (Neurotic Anxiety States)

##

## MEDLINE (via PubMed) (n=226)

| #1Diabetes Mellitus, Type 1 [MeSH] + Entry Terms | (((((((((((((((((((((((((((((((((((((((("diabetes mellitus, type 1"[MeSH Terms] OR "type 1 diabetes mellitus"[All Fields]) OR ("autoimmune"[All Fields] AND "diabetes"[All Fields])) OR "autoimmune diabetes"[All Fields]) OR ((("diabetes mellitus, type 1"[MeSH Terms] OR "type 1 diabetes mellitus"[All Fields]) OR (("brittle"[All Fields] AND "diabetes"[All Fields]) AND "mellitus"[All Fields])) OR "brittle diabetes mellitus"[All Fields])) OR (("diabetes mellitus, type 1"[MeSH Terms] OR "type 1 diabetes mellitus"[All Fields]) OR "iddm"[All Fields])) OR ((("diabetes mellitus, type 1"[MeSH Terms] OR "type 1 diabetes mellitus"[All Fields]) OR ((("insulin"[All Fields] AND "dependent"[All Fields]) AND "diabetes"[All Fields]) AND "mellitus"[All Fields])) OR "insulin dependent diabetes mellitus"[All Fields])) OR (("diabetes mellitus, type 1"[MeSH Terms] OR "type 1 diabetes mellitus"[All Fields]) OR "insulin dependent diabetes mellitus 1"[All Fields])) OR ((("diabetes mellitus, type 1"[MeSH Terms] OR "type 1 diabetes mellitus"[All Fields]) OR ((("insulin"[All Fields] AND "dependent"[All Fields]) AND "diabetes"[All Fields]) AND "mellitus"[All Fields])) OR "insulin dependent diabetes mellitus"[All Fields])) OR (("diabetes mellitus, type 1"[MeSH Terms] OR "type 1 diabetes mellitus"[All Fields]) OR "insulin dependent diabetes mellitus 1"[All Fields])) OR ((("diabetes mellitus, type 1"[MeSH Terms] OR "type 1 diabetes mellitus"[All Fields]) OR (("juvenile"[All Fields] AND "onset"[All Fields]) AND "diabetes"[All Fields])) OR "juvenile onset diabetes"[All Fields])) OR ((("diabetes mellitus, type 1"[MeSH Terms] OR "type 1 diabetes mellitus"[All Fields]) OR ((("juvenile"[All Fields] AND "onset"[All Fields]) AND "diabetes"[All Fields]) AND "mellitus"[All Fields])) OR "juvenile onset diabetes mellitus"[All Fields])) OR ((("diabetes mellitus, type 1"[MeSH Terms] OR "type 1 diabetes mellitus"[All Fields]) OR (("juvenile"[All Fields] AND "onset"[All Fields]) AND "diabetes"[All Fields])) OR "juvenile onset diabetes"[All Fields])) OR ((("diabetes mellitus, type 1"[MeSH Terms] OR "type 1 diabetes mellitus"[All Fields]) OR ((("juvenile"[All Fields] AND "onset"[All Fields]) AND "diabetes"[All Fields]) AND "mellitus"[All Fields])) OR "juvenile onset diabetes mellitus"[All Fields])) OR ((("diabetes mellitus, type 1"[MeSH Terms] OR "type 1 diabetes mellitus"[All Fields]) OR ((("ketosis"[All Fields] AND "prone"[All Fields]) AND "diabetes"[All Fields]) AND "mellitus"[All Fields])) OR "ketosis prone diabetes mellitus"[All Fields])) OR ((("diabetes mellitus, type 1"[MeSH Terms] OR "type 1 diabetes mellitus"[All Fields]) OR ((("ketosis"[All Fields] AND "prone"[All Fields]) AND "diabetes"[All Fields]) AND "mellitus"[All Fields])) OR "ketosis prone diabetes mellitus"[All Fields])) OR ((("diabetes mellitus, type 1"[MeSH Terms] OR "type 1 diabetes mellitus"[All Fields]) OR ((("sudden"[All Fields] AND "onset"[All Fields]) AND "diabetes"[All Fields]) AND "mellitus"[All Fields])) OR "sudden onset diabetes mellitus"[All Fields])) OR ((("diabetes mellitus, type 1"[MeSH Terms] OR "type 1 diabetes mellitus"[All Fields]) OR ((("sudden"[All Fields] AND "onset"[All Fields]) AND "diabetes"[All Fields]) AND "mellitus"[All Fields])) OR "sudden onset diabetes mellitus"[All Fields])) OR (("diabetes mellitus, type 1"[MeSH Terms] OR "type 1 diabetes mellitus"[All Fields]) OR "type 1 diabetes"[All Fields])) OR ("diabetes mellitus, type 1"[MeSH Terms] OR "type 1 diabetes mellitus"[All Fields])) OR (("diabetes mellitus, type 1"[MeSH Terms] OR "type 1 diabetes mellitus"[All Fields]) OR "type i diabetes mellitus"[All Fields])) |
| --- | --- |
| #2Diabetes Mellitus, Type 2  [MeSH] + Entry Terms | ((("diabetes mellitus, type 2"[MeSH Terms] OR "type 2 diabetes mellitus"[All Fields]) OR ((("adult"[All Fields] AND "onset"[All Fields]) AND "diabetes"[All Fields]) AND "mellitus"[All Fields])) OR "adult onset diabetes mellitus"[All Fields])) OR ((("diabetes mellitus, type 2"[MeSH Terms] OR "type 2 diabetes mellitus"[All Fields]) OR ((("adult"[All Fields] AND "onset"[All Fields]) AND "diabetes"[All Fields]) AND "mellitus"[All Fields])) OR "adult onset diabetes mellitus"[All Fields])) OR ((("diabetes mellitus, type 2"[MeSH Terms] OR "type 2 diabetes mellitus"[All Fields]) OR ((("ketosis"[All Fields] AND "resistant"[All Fields]) AND "diabetes"[All Fields]) AND "mellitus"[All Fields])) OR "ketosis resistant diabetes mellitus"[All Fields])) OR ((("diabetes mellitus, type 2"[MeSH Terms] OR "type 2 diabetes mellitus"[All Fields]) OR ((("ketosis"[All Fields] AND "resistant"[All Fields]) AND "diabetes"[All Fields]) AND "mellitus"[All Fields])) OR "ketosis resistant diabetes mellitus"[All Fields])) OR ((("diabetes mellitus, type 2"[MeSH Terms] OR "type 2 diabetes mellitus"[All Fields]) OR (("maturity"[All Fields] AND "onset"[All Fields]) AND "diabetes"[All Fields])) OR "maturity onset diabetes"[All Fields])) OR ((("diabetes mellitus, type 2"[MeSH Terms] OR "type 2 diabetes mellitus"[All Fields]) OR ((("maturity"[All Fields] AND "onset"[All Fields]) AND "diabetes"[All Fields]) AND "mellitus"[All Fields])) OR "maturity onset diabetes mellitus"[All Fields])) OR ((("diabetes mellitus, type 2"[MeSH Terms] OR "type 2 diabetes mellitus"[All Fields]) OR (("maturity"[All Fields] AND "onset"[All Fields]) AND "diabetes"[All Fields])) OR "maturity onset diabetes"[All Fields])) OR ((("diabetes mellitus, type 2"[MeSH Terms] OR "type 2 diabetes mellitus"[All Fields]) OR ((("maturity"[All Fields] AND "onset"[All Fields]) AND "diabetes"[All Fields]) AND "mellitus"[All Fields])) OR "maturity onset diabetes mellitus"[All Fields])) OR (("diabetes mellitus, type 2"[MeSH Terms] OR "type 2 diabetes mellitus"[All Fields]) OR "mody"[All Fields])) OR ((("diabetes mellitus, type 2"[MeSH Terms] OR "type 2 diabetes mellitus"[All Fields]) OR "niddm"[All Fields]) OR "niddms"[All Fields])) OR ((("diabetes mellitus, type 2"[MeSH Terms] OR "type 2 diabetes mellitus"[All Fields]) OR (((("non"[All Fields] AND "insulin"[All Fields]) AND "dependent"[All Fields]) AND "diabetes"[All Fields]) AND "mellitus"[All Fields])) OR "non insulin dependent diabetes mellitus"[All Fields])) OR ((("diabetes mellitus, type 2"[MeSH Terms] OR "type 2 diabetes mellitus"[All Fields]) OR ((("noninsulin"[All Fields] AND "dependent"[All Fields]) AND "diabetes"[All Fields]) AND "mellitus"[All Fields])) OR "noninsulin dependent diabetes mellitus"[All Fields])) OR ((("diabetes mellitus, type 2"[MeSH Terms] OR "type 2 diabetes mellitus"[All Fields]) OR ((("noninsulin"[All Fields] AND "dependent"[All Fields]) AND "diabetes"[All Fields]) AND "mellitus"[All Fields])) OR "noninsulin dependent diabetes mellitus"[All Fields])) OR ((("diabetes mellitus, type 2"[MeSH Terms] OR "type 2 diabetes mellitus"[All Fields]) OR (((("non"[All Fields] AND "insulin"[All Fields]) AND "dependent"[All Fields]) AND "diabetes"[All Fields]) AND "mellitus"[All Fields])) OR "non insulin dependent diabetes mellitus"[All Fields])) OR (("diabetes mellitus, type 2"[MeSH Terms] OR "type 2 diabetes mellitus"[All Fields]) OR ((("slow"[All Fields] AND "onset"[All Fields]) AND "diabetes"[All Fields]) AND "mellitus"[All Fields]))) OR (("diabetes mellitus, type 2"[MeSH Terms] OR "type 2 diabetes mellitus"[All Fields]) OR ((("slow"[All Fields] AND "onset"[All Fields]) AND "diabetes"[All Fields]) AND "mellitus"[All Fields]))) OR ((("diabetes mellitus, type 2"[MeSH Terms] OR "type 2 diabetes mellitus"[All Fields]) OR (("stable"[All Fields] AND "diabetes"[All Fields]) AND "mellitus"[All Fields])) OR "stable diabetes mellitus"[All Fields])) OR (("diabetes mellitus, type 2"[MeSH Terms] OR "type 2 diabetes mellitus"[All Fields]) OR "type 2 diabetes"[All Fields])) OR ("diabetes mellitus, type 2"[MeSH Terms] OR "type 2 diabetes mellitus"[All Fields])) OR ((("diabetes mellitus, type 2"[MeSH Terms] OR "type 2 diabetes mellitus"[All Fields]) OR ((("type"[All Fields] AND "ii"[All Fields]) AND "diabetes"[All Fields]) AND "mellitus"[All Fields])) OR "type ii diabetes mellitus"[All Fields])) |
| #3Depression [MeSH] + Entry Terms | ((((((((((((((((((((((((((((((((((((((((("depressed"[All Fields] OR "depression"[MeSH Terms]) OR "depression"[All Fields]) OR "depressions"[All Fields]) OR "depression s"[All Fields]) OR "depressive disorder"[MeSH Terms]) OR ("depressive"[All Fields] AND "disorder"[All Fields])) OR "depressive disorder"[All Fields]) OR "depressivity"[All Fields]) OR "depressive"[All Fields]) OR "depressively"[All Fields]) OR "depressiveness"[All Fields]) OR "depressives"[All Fields]) (((((((((((("depressed"[All Fields] OR "depression"[MeSH Terms]) OR "depression"[All Fields]) OR "depressions"[All Fields]) OR "depression s"[All Fields]) OR "depressive disorder"[MeSH Terms]) OR ("depressive"[All Fields] AND "disorder"[All Fields])) OR "depressive disorder"[All Fields]) OR "depressivity"[All Fields]) OR "depressive"[All Fields]) OR "depressively"[All Fields]) OR "depressiveness"[All Fields]) OR "depressives"[All Fields])) OR ((("depression"[MeSH Terms] OR "depression"[All Fields]) OR ("depressive"[All Fields] AND "symptom"[All Fields])) OR "depressive symptom"[All Fields])) OR ((("depression"[MeSH Terms] OR "depression"[All Fields]) OR ("depressive"[All Fields] AND "symptoms"[All Fields])) OR "depressive symptoms"[All Fields])) OR ((("depression"[MeSH Terms] OR "depression"[All Fields]) OR ("emotional"[All Fields] AND "depression"[All Fields])) OR "emotional depression"[All Fields])) OR (("depression"[MeSH Terms] OR "depression"[All Fields]) OR ("emotional"[All Fields] AND "depressions"[All Fields]))) |
| #4Depressive Disorder [MeSH] + Entry Terms | (("depressive disorder"[MeSH Terms] OR ("depressive"[All Fields] AND "disorder"[All Fields])) OR "depressive disorder"[All Fields])) OR (((("depressive disorder"[MeSH Terms] OR ("depressive"[All Fields] AND "disorder"[All Fields])) OR "depressive disorder"[All Fields]) OR ("depressive"[All Fields] AND "disorders"[All Fields])) OR "depressive disorders"[All Fields])) OR (((("depressive disorder"[MeSH Terms] OR ("depressive"[All Fields] AND "disorder"[All Fields])) OR "depressive disorder"[All Fields]) OR ("depressive"[All Fields] AND "neuroses"[All Fields])) OR "depressive neuroses"[All Fields])) OR (((("depressive disorder"[MeSH Terms] OR ("depressive"[All Fields] AND "disorder"[All Fields])) OR "depressive disorder"[All Fields]) OR ("depressive"[All Fields] AND "neurosis"[All Fields])) OR "depressive neurosis"[All Fields])) OR (((("depressive disorder"[MeSH Terms] OR ("depressive"[All Fields] AND "disorder"[All Fields])) OR "depressive disorder"[All Fields]) OR ("depressive"[All Fields] AND "syndrome"[All Fields])) OR "depressive syndrome"[All Fields])) OR (((("depressive disorder"[MeSH Terms] OR ("depressive"[All Fields] AND "disorder"[All Fields])) OR "depressive disorder"[All Fields]) OR ("depressive"[All Fields] AND "syndromes"[All Fields])) OR "depressive syndromes"[All Fields])) OR (((("depressive disorder"[MeSH Terms] OR ("depressive"[All Fields] AND "disorder"[All Fields])) OR "depressive disorder"[All Fields]) OR ("endogenous"[All Fields] AND "depression"[All Fields])) OR "endogenous depression"[All Fields])) OR (((("depressive disorder"[MeSH Terms] OR ("depressive"[All Fields] AND "disorder"[All Fields])) OR "depressive disorder"[All Fields]) OR ("endogenous"[All Fields] AND "depressions"[All Fields])) OR "endogenous depressions"[All Fields])) OR (((("depressive disorder"[MeSH Terms] OR ("depressive"[All Fields] AND "disorder"[All Fields])) OR "depressive disorder"[All Fields]) OR "melancholia"[All Fields]) OR "melancholias"[All Fields])) OR (((("depressive disorder"[MeSH Terms] OR ("depressive"[All Fields] AND "disorder"[All Fields])) OR "depressive disorder"[All Fields]) OR "melancholia"[All Fields]) OR "melancholias"[All Fields])) OR (((("depressive disorder"[MeSH Terms] OR ("depressive"[All Fields] AND "disorder"[All Fields])) OR "depressive disorder"[All Fields]) OR ("neurotic"[All Fields] AND "depression"[All Fields])) OR "neurotic depression"[All Fields])) OR (((("depressive disorder"[MeSH Terms] OR ("depressive"[All Fields] AND "disorder"[All Fields])) OR "depressive disorder"[All Fields]) OR ("neurotic"[All Fields] AND "depressions"[All Fields])) OR "neurotic depressions"[All Fields])) OR (((("depressive disorder"[MeSH Terms] OR ("depressive"[All Fields] AND "disorder"[All Fields])) OR "depressive disorder"[All Fields]) OR ("unipolar"[All Fields] AND "depression"[All Fields])) OR "unipolar depression"[All Fields])) OR (((("depressive disorder"[MeSH Terms] OR ("depressive"[All Fields] AND "disorder"[All Fields])) OR "depressive disorder"[All Fields]) OR ("unipolar"[All Fields] AND "depressions"[All Fields])) OR "unipolar depressions"[All Fields])) |
| #5Anxiety [MeSH] + Entry Terms | ((("anxiety"[MeSH Terms] OR "anxiety"[All Fields]) OR "anxieties"[All Fields]) OR "anxiety s"[All Fields])) OR ((("anxiety"[MeSH Terms] OR "anxiety"[All Fields]) OR "hypervigilance"[All Fields]) OR "hypervigilant"[All Fields])) OR (("anxiety"[MeSH Terms] OR "anxiety"[All Fields]) OR "nervousness"[All Fields])) OR ((("anxiety"[MeSH Terms] OR "anxiety"[All Fields]) OR ("social"[All Fields] AND "anxieties"[All Fields])) OR "social anxieties"[All Fields])) OR ((("anxiety"[MeSH Terms] OR "anxiety"[All Fields]) OR ("social"[All Fields] AND "anxieties"[All Fields])) OR "social anxieties"[All Fields])) OR ((("anxiety"[MeSH Terms] OR "anxiety"[All Fields]) OR ("social"[All Fields] AND "anxiety"[All Fields])) OR "social anxiety"[All Fields])) |
| #6Anxiety Disorders [MeSH] + Entry Terms | (((("anxiety disorders"[MeSH Terms] OR ("anxiety"[All Fields] AND "disorders"[All Fields])) OR "anxiety disorders"[All Fields]) OR ("anxiety"[All Fields] AND "disorder"[All Fields])) OR "anxiety disorder"[All Fields])) OR (("anxiety disorders"[MeSH Terms] OR ("anxiety"[All Fields] AND "disorders"[All Fields])) OR "anxiety disorders"[All Fields])) OR (((("anxiety disorders"[MeSH Terms] OR ("anxiety"[All Fields] AND "disorders"[All Fields])) OR "anxiety disorders"[All Fields]) OR (("neurotic"[All Fields] AND "anxiety"[All Fields]) AND "states"[All Fields])) OR "neurotic anxiety states"[All Fields])) OR (((("anxiety disorders"[MeSH Terms] OR ("anxiety"[All Fields] AND "disorders"[All Fields])) OR "anxiety disorders"[All Fields]) OR (("neurotic"[All Fields] AND "anxiety"[All Fields]) AND "states"[All Fields])) OR "neurotic anxiety states"[All Fields])) |
| #7 | **#1 OR #2 AND #3 OR #4 OR #5 OR #6** |
| #8 | **Systematic Review [Publication type]** |
| #9 | **#7 AND #8** |

**LILACS (via Virtual Health Library) (n=5)**

| #1 | ((tw:((diabetes mellitus) OR (hyperglycemia) OR (glucose intolerance) OR (type 1 diabetes mellitus) OR (autoimmune diabetes) OR (brittle diabetes mellitus) OR (brittle diabetes mellitu) OR (insulin dependent diabetes mellitus) OR (insulin-dependent diabetes mellitus) OR (insulin-dependent diabetes mellitus 1) OR (juvenile onset diabetes mellitus) OR (juvenile-onset diabetes mellitus) OR (ketosis prone diabetes mellitus) OR (ketosis-prone diabetes mellitus) OR (sudden onset diabetes mellitus) OR (sudden-onset diabetes mellitus) OR (type i diabetes mellitus) OR (autoimmune diabetes) OR (juvenile-onset diabetes) OR (type 1 diabetes) OR (iddm) OR (insulin dependent diabetes mellitus 1) OR (insulin-dependent diabetes mellitus) OR (insulin-dependent diabetes mellitus 1) OR (juvenile onset diabetes) OR (juvenile-onset diabetes) OR (juvenile-onset diabetes mellitus) OR (ketosis-prone diabetes mellitus) OR (sudden-onset diabetes mellitus) OR (type 1 diabetes) OR (type 1 diabetes mellitus) OR (adult-onset diabetes mellitus) OR (adult onset diabetes mellitus) OR (ketosis resistant diabetes mellitus) OR (ketosis-resistant diabetes mellitus) OR (maturity onset diabetes mellitus) OR (maturity-onset diabetes mellitus) OR (maturity onset diabetes) OR (maturity-onset diabetes) OR (non insulin dependent diabetes mellitus) OR (non-insulin-dependent diabetes mellitus) OR (noninsulin dependent diabetes mellitus) OR (noninsulin-dependent diabetes mellitus) OR (slow onset diabetes mellitus) OR (slow-onset diabetes mellitus) OR (stable diabetes mellitus) OR (type ii diabetes mellitus) OR (mody) OR (niddm) OR (slow-onset diabetes mellitus) OR (stable diabetes mellitus) OR (type 2 diabetes) OR (type 2 diabetes mellitus))) |
| --- | --- |
| #2 | (tw:((depression) OR (depressions) OR (depressive symptom) OR (depressive symptoms) OR (emotional depression) OR (emotional depressions) OR (depressive disorder) OR (depressive disorders) OR (depressive neuroses) OR (depressive neurosis) OR (depressive syndrome) OR (depressive syndromes) OR (endogenous depression) OR (endogenous depressions) OR (melancholia) OR (melancholias) OR (neurotic depression) OR (neurotic depressions) OR (unipolar depression) OR (unipolar depressions) OR (anxiety) OR (hypervigilance) OR (nervousness) OR (social anxieties) OR (social anxiety) OR (anxiety disorders) OR (anxiety disorder) OR (anxiety neuroses) OR (neurotic anxiety state) OR (neurotic anxiety states))) |
| #3 | (tw:( systematic review))) |
| #4 | (db:("LILACS") AND type_of_study:("systematic_reviews")) |
| #5 | #1 AND #2 AND #3 AND #4 |

**Cochrane Library (n=53)**

| #1 | (Diabetes Complications) OR (Diabetes Mellitus, Type 1) OR (Diabetes Mellitus, Type 2) |
| --- | --- |
| #2 | (Depression) OR (Depressive Disorder) OR (Anxiety) OR (Anxiety Disorders) |
| #3 | #1 AND #2 in Cochrane Reviews with Child Health, Common Mental Disorders, Metabolic and Endocrine Disorders, Complementary Medicine, Public Health in Cochrane Groups |

**Web of Science (n=381)**

| #1 | TS=((Autoimmune Diabetes)  OR (Brittle Diabetes Mellitus)  OR (IDDM)  OR (Insulin Dependent Diabetes Mellitus)  OR (Insulin Dependent Diabetes Mellitus 1)  OR (Insulin-Dependent Diabetes Mellitus)  OR (Insulin-Dependent Diabetes Mellitus 1)  OR (Juvenile Onset Diabetes)  OR (Juvenile Onset Diabetes Mellitus)  OR (Juvenile-Onset Diabetes)  OR (Juvenile-Onset Diabetes Mellitus)  OR (Ketosis Prone Diabetes Mellitus)  OR (Ketosis-Prone Diabetes Mellitus)  OR (Sudden Onset Diabetes Mellitus)  OR (Sudden-Onset Diabetes Mellitus)  OR (Type 1 Diabetes)  OR (Type 1 Diabetes Mellitus)  OR (Type I Diabetes Mellitus)  OR (Adult Onset Diabetes Mellitus)  OR (Adult-Onset Diabetes Mellitus)  OR (Ketosis Resistant Diabetes Mellitus)  OR (Ketosis-Resistant Diabetes Mellitus)  OR (Maturity Onset Diabetes)  OR (Maturity Onset Diabetes Mellitus)  OR (Maturity-Onset Diabetes)  OR (Maturity-Onset Diabetes Mellitus)  OR (MODY)  OR (NIDDM)  OR (Non Insulin Dependent Diabetes Mellitus)  OR (Noninsulin Dependent Diabetes Mellitus)  OR (Noninsulin-Dependent Diabetes Mellitus)  OR (Non-Insulin-Dependent Diabetes Mellitus)  OR (Slow Onset Diabetes Mellitus)  OR (Slow-Onset Diabetes Mellitus)  OR (Stable Diabetes Mellitus)  OR (Type 2 Diabetes)  OR (Type 2 Diabetes Mellitus)  OR (Type II Diabetes Mellitus)) |
| --- | --- |
| #2 | TS=((Depression) OR (Depressions) OR (Depressive Symptom) OR (Depressive Symptoms) OR (Emotional Depression) OR (Emotional Depressions) OR (Depressive Disorder) OR (Depressive Disorders) OR (Depressive Neuroses) OR (Depressive Neurosis) OR (Depressive Syndrome) OR (Depressive Syndromes) OR (Endogenous Depression) OR (Endogenous Depressions) OR (Melancholia) OR (Melancholias) OR (Neurotic Depression) OR (Neurotic Depressions) OR (Unipolar Depression) OR (Unipolar Depressions) OR (Anxiety) OR (Hypervigilance) OR (Nervousness) OR (Social Anxieties) OR (Social Anxieties) OR (Social Anxiety) 0R (Anxiety Disorder) OR (Anxiety Disorders) OR (Neurotic Anxiety State) OR (Neurotic Anxiety States)) |
| #3 | TS=(systematic review) |
| #4 | #3  AND #2  AND #1 |

**EMBASE (n=681)**

| #1 | 'non insulin dependent diabetes mellitus'/exp AND [embase]/lim |
| --- | --- |
| #2 | 'insulin dependent diabetes mellitus'/exp AND [embase]/lim |
| #3 | 'depression'/exp AND [embase]/lim |
| #4 | 'major depression'/exp AND [embase]/lim |
| #5 | 'depressive disorder'/exp AND [embase]/lim |
| #6 | 'endogenous depression'/exp AND [embase]/lim |
| #7 | 'melancholia'/exp AND [embase]/lim |
| #8 | 'anxiety disorder'/exp AND [embase]/lim |
| #9 | 'anxiety'/exp AND [embase]/lim |
| #10 | 'nervousness'/exp AND [embase]/lim |
| #11 | #1 OR #2 |
| #12 | #3 OR #4 OR #5 OR #6 OR #7 OR #8 OR #9 OR #10 |
| #13 | #11 AND #12 AND ([cochrane review]/lim OR [systematic review]/lim OR [meta analysis]/lim) |
