# Supplementary material for: Interventions for depression and anxiety among people with diabetes mellitus: Review of systematic reviews
Source: PLoS One. 2023 Feb 9;18(2):e0281376. doi: 10.1371/journal.pone.0281376 (PMC9910656; doi:10.1371/journal.pone.0281376)
Supplement: Attachment — Submitted filename: Response to Reviewers.pdf [file pone.0281376.s010.pdf]

PONE-D-22-05027

Interventions for depression and anxiety among people with diabetes mellitus: an overview of systematic reviews and meta-analyses

Dear Dr. Bergamaschi,

Thank you for submitting your manuscript to PLOS ONE. After careful consideration, we feel that it has merit but does not fully meet PLOS ONE's publication criteria as it currently stands. Therefore, we invite you to submit a revised version of the manuscript that addresses the points raised during the review process. Please note that we have only been able to secure a single reviewer to assess your manuscript. We are issuing a decision on your manuscript at this point to prevent further delays in the evaluation of your manuscript. Please be aware that the editor who handles your revised manuscript might find it necessary to invite additional reviewers to assess this work once the revised manuscript is submitted. However, we will aim to proceed on the basis of this single review if possible. The reviewer raised a number of concerns that need attention. They request additional information on methodological aspects of the study such as finer details report age range, inclusion criteria of individual reviews, and diabetes duration. Could you please revise the manuscript to carefully address the concerns raised?

September, 5nd 2022

Dear editor Katrien Janin

Thank you for giving us another opportunity to answer the comments made by reviewer in our manuscript entitled: "Critical appraisal of clinical practice guidelines for the treatment of urinary incontinence: a systematic review" (Ref.: Ms. No. IUJO-D-20-00791).

We have inserted additional information on methodological aspects of the study such as details (age range, inclusion criteria of individual reviews, diabetes duration). The most reviews did not report diabetes. The studies that reported this information are highlighted in the "Characteristics of the study" table. We too insert study's minimal underlying data as "supporting information file" (S3 Appendix).

We are looking forward to hearing from you.

Sincerely,

Dr. Cristiane de Cássia Bergamaschi (University of Sorocaba - Sorocaba/SP, Brazil)

Reviewers' comments:

1. Is the manuscript technically sound, and do the data support the conclusions? The manuscript must describe a technically sound piece of scientific research with data that supports the conclusions. Experiments must have been conducted rigorously, with appropriate controls, replication, and sample sizes. The conclusions must be drawn appropriately based on the data presented.

Reviewer #1: Yes

2. Has the statistical analysis been performed appropriately and rigorously?

Reviewer #1: N/A

3. Have the authors made all data underlying the findings in their manuscript fully available? The PLOS Data policy requires authors to make all data underlying the findings described in their manuscript fully available without restriction, with rare exception (please refer to the Data Availability Statement in the manuscript PDF file). The data should be provided as part of the manuscript or its supporting information, or deposited to a public repository. For example, in addition to summary statistics, the data points behind means, medians and variance measures should be available. If there are restrictions on publicly sharing data—e.g. participant privacy or use of data from a third party—those must be specified.

Reviewer #1: Yes

4. Is the manuscript presented in an intelligible fashion and written in standard English? PLOS ONE does not copyedit accepted manuscripts, so the language in submitted articles must be clear, correct, and unambiguous. Any typographical or grammatical errors should be corrected at revision, so please note any specific errors here.

Reviewer #1: Yes

Dear Reviewer

Thank you for giving us another opportunity to answer the comments made by reviewer in our manuscript. We answer all comments and remain at your disposal if further adjustments are necessary.

1. This is an important review of reviews of interventions for depression and anxiety for people with diabetes. This area is well researched so it's important to bring together all the literature in one place. I would expect something like this to be called 'a review of reviews. Is it a review of meta-analyses if you are narratively synthesising the information? Might be appropriate to use the vote count method (which would be straightforward based on what you have achieved already), see SWiM guidelines: https://www.bmj.com/content/368/bmj.l6890

Answer: Dear reviewer, thank you for the suggestions that contributed to the improvement of the manuscript. We agree with the change in the writing of the study design "overview of systematic reviews and meta-analyses". We carried out a search on Prospero and the most of the protocols cite this type of study as: "review of systematic reviews". Then, we modified the title, but if the reviewer deems another adjustment necessary, we remain at your disposal.

2. Abstract

Clarify 'standard interventions'- do you mean usual care? 'Among others' is too vague- please specify. If short on words, only report primary outcomes in abstract. 'Reviewers, in pairs and independently' is unclear. Please expand on 'narrative synthesis'- did you tabulate results, organize by any group (I assume intervention type?)?

Answer: Dear reviewer, we accept all suggestions and we have rewritten the parts of the abstract in which changes were requested.

3. Introduction

Diabetes is not a 'disease', it is a long-term health condition. Avoid terms such as 'suffering' and 'control' and 'patients', see language matters guidance: https://www.england.nhs.uk/wp-content/uploads/2018/06/language-matters.pdf

Answer: We accept the suggestions and adapt most of the text with the suggested words. We have changed the term "glycemic control" to "glycemic values" in most of the text. "Glycemic control" was a term well used by the included reviews and sometimes it was necessary to keep it. However, we remain at your disposal if any other adjustments are necessary.

4. Avoiding multiple prevalence data, with different stats representing same information (i.e. in first paragraph). *Regardless of their age' is an odd phrase. Are you looking to compare adolescent's vs adults? Consider* phraseology here. It looks like you go on to only review studies with adults- so you might not need this clause in the research aim/question.

Answer: We have made the adjustments as requested.

5. Often unclear whether data and studies involve T1D and/or T2D. I would expect to see stronger justification for including both T1D and T2D, and not just focusing on one type. You allude to safety in your aims, but this isn't introduced in the background research- please provide rational for this inclusion.

Answer: We included studies with both type 1 and type 2 diabetes since the literature have demonstrated that psychological disorders are present in individuals with both diseases. When

we read the full text of the included reviews, most of the reviews included both the type 1 and type 2 diabetes population, as well as the meta-analyses pooled information from both populations. Of the 13 reviews, 9 included both populations. We sought to make this information clearer in the “Introduction” and “Discussion” of the study.

Little information is available in the literature regarding the safety of interventions. In our results, this information was restricted to report of adverse reactions to antidepressants. Since safety was a secondary outcome in our study, we chose not to highlight it in the introduction, but we inserted this information in “Results” and pointed out this limitation of the studies in “Discussion”.

6. Methods

Ethics statement not need for SR. Misplaced bracket in line 123. Again, clarify ‘Pairs of reviewers (RF and IM) and independently’

Answer: We have made the adjustments as requested.

7. Outcomes should be more specific in eligibility criteria. Could consider adding a table to represent inclusion/exclusion criteria.

Answer: We have inserted more detailed information on outcomes in “Outcomes evaluated”, since the table would be disproportionate with more information on inclusion than exclusion criteria. However, we remain at your disposal if further adjustments are necessary.

8. How many articles were requested from authors? How many did you obtain from this process? (This might belong in results section)

Answer: We enter this information in the “Results” as requested.

9. Results

The study characteristics section seems quite limited. I would expect to see a PCIOs break down as reported in methods. E.g. Can you finer details report age range, inclusion criteria of individual reviews, diabetes duration etc. It would be useful to see comment on the measures used for depression and anxiety across reviews.

Answer: We made some adjustments in “Results”. We placed the “psychiatric illness” column before “intervention” column to bring it closer to population information. We highlighted the duration of diabetes disease in the footnote, as only two reviews provided this information. The reviews used different tools and scales to measure anxiety and depression and this information is available in S3 Appendix (Supporting information file).
