# Supplementary material for: Interventions for depression and anxiety among people with diabetes mellitus: Review of systematic reviews
Source: PLoS One. 2023 Feb 9;18(2):e0281376. doi: 10.1371/journal.pone.0281376 (PMC9910656; doi:10.1371/journal.pone.0281376)
Supplement: Attachment — Submitted filename: Response to Reviewers.docx [file pone.0281376.s011.docx]

Dear Dr. Bergamaschi,
Thank you for submitting your manuscript to PLOS ONE. After careful consideration, we feel that it has merit but does not fully meet PLOS ONE’s publication criteria as it currently stands. Therefore, we invite you to submit a revised version of the manuscript that addresses the points raised during the review process.
Your manuscript has been assessed by an additional reviewer whose report can be found below. As you will see from the comments, there remain some concerns which should be addressed before your manuscript is suitable for publication. In particular, we request that you update your search to include the most recent studies. 
Please submit your revised manuscript by Jan 02 2023 11:59PM. If you will need more time than this to complete your revisions, please reply to this message or contact the journal office at [plosone@plos.org](mailto:plosone@plos.org). When you're ready to submit your revision, log on to https://www.editorialmanager.com/pone/ and select the 'Submissions Needing Revision' folder to locate your manuscript file.
Please include the following items when submitting your revised manuscript:

- A rebuttal letter that responds to each point raised by the academic editor and reviewer(s). You should upload this letter as a separate file labeled 'Response to Reviewers'.
- A marked-up copy of your manuscript that highlights changes made to the original version. You should upload this as a separate file labeled 'Revised Manuscript with Track Changes'.
- An unmarked version of your revised paper without tracked changes. You should upload this as a separate file labeled 'Manuscript'.

If you would like to make changes to your financial disclosure, please include your updated statement in your cover letter. Guidelines for resubmitting your figure files are available below the reviewer comments at the end of this letter.
We look forward to receiving your revised manuscript.

Kind regards,

Dr Senior Editor
PLOS ONE

December, 14^nd^ 2022

**Dear editor Joseph Donlan**

Thank you for giving us the opportunity to answer the comments made by reviewer in our manuscript entitled: “Interventions for depression and anxiety among people with diabetes mellitus: an overview of systematic reviews and meta-analyses" (PONE-D-22-05027).

We have inserted all the information requested by the reviewer and updated the bibliographic search in the databases carried out in December 2022. The authors look forward to a response as soon as possible.

We are looking forward to hearing from you.

Sincerely,

Dr. Cristiane de Cássia Bergamaschi

University of Sorocaba - Sorocaba/SP, Brazil

**Reviewers' comments:**

1. If the authors have adequately addressed your comments raised in a previous round of review and you feel that this manuscript is now acceptable for publication, you may indicate that here to bypass the “Comments to the Author” section, enter your conflict-of-interest statement in the “Confidential to Editor” section, and submit your "Accept" recommendation.

Reviewer #2: (No Response)

2. Is the manuscript technically sound, and do the data support the conclusions?
The manuscript must describe a technically sound piece of scientific research with data that supports the conclusions. Experiments must have been conducted rigorously, with appropriate controls, replication, and sample sizes. The conclusions must be drawn appropriately based on the data presented. Reviewer #2: Partly

**Answer:** Dear reviewer, thank you for the suggestions that contributed to the improvement of the manuscript. We redid the search strategy and identified 10 systematic reviews with potential to be included in the study. After reading of the full texts, none of them met the eligibility criteria for our study. These reviews were included in the list of excluded studies (S2 Appendix) and the references are highlighted “in blue”. We remain at your disposal if any other adjustment will be necessary.

3. Has the statistical analysis been performed appropriately and rigorously?

Reviewer #2: (No Response)

4. Have the authors made all data underlying the findings in their manuscript fully available?
The [PLOS Data policy](https://nam02.safelinks.protection.outlook.com/?url=http%3A%2F%2Fwww.plosone.org%2Fstatic%2Fpolicies.action%23sharing&data=05%7C01%7Ccristiane.motta%40prof.uniso.br%7C86061c2ddff64aa9e3d508dacccb0112%7Cd987bd71a5f144e3bb25e111ab6ffa13%7C0%7C0%7C638047469532593134%7CUnknown%7CTWFpbGZsb3d8eyJWIjoiMC4wLjAwMDAiLCJQIjoiV2luMzIiLCJBTiI6Ik1haWwiLCJXVCI6Mn0%3D%7C3000%7C%7C%7C&sdata=J%2FJpBnw9Tgrjrbum1Ri6RBk%2B9lIKZ9FtCZgqkkoEUy8%3D&reserved=0) requires authors to make all data underlying the findings described in their manuscript fully available without restriction, with rare exception (please refer to the Data Availability Statement in the manuscript PDF file). The data should be provided as part of the manuscript or its supporting information, or deposited to a public repository. For example, in addition to summary statistics, the data points behind means, medians and variance measures should be available. If there are restrictions on publicly sharing data—e.g. participant privacy or use of data from a third party—those must be specified.

Reviewer #2: No

**Answer:** All the study data is available in an excel spreadsheet. This file is named as “S3 Appendix”.

5. Is the manuscript presented in an intelligible fashion and written in standard English?
PLOS ONE does not copyedit accepted manuscripts, so the language in submitted articles must be clear, correct, and unambiguous. Any typographical or grammatical errors should be corrected at revision, so please note any specific errors here. Reviewer #2: Yes

6. Review Comments to the Author: Dear Authors, unfortunately, there are several fundamental errors.

1- The last search is July 2021, which means you lost several related studies during more than one year ago. On the other hand, it is currently published some meta-analyses in the topic: Mersha AG, Tollosa DN, Bagade T, Eftekhari P. A bidirectional relationship between diabetes mellitus and anxiety: A systematic review and meta-analysis. J Psychosom Res. 2022 Nov;162:110991. doi: 10.1016/j.jpsychores.2022.110991. Association between social supports and depression among patients with diabetes mellitus in Ethiopia: a systematic review and meta-analysis. BMJ Open. 2022 May 11;12(5):e061801.

**Answer:** As requested by the reviewer, we redid the search strategy and identified 10 potentially eligible systematic reviews. After selecting the studies based on reading the full texts, none of them met the eligibility criteria. The S2 Appendix contain the list of excluded studies along with the justifications and information highlighted in blue. The studies cited by the reviewer were not included in “results”, since they are reviews that reported disease prevalence data. We inserted one of reviews in “introduction”.

2- At the first should be clear definition of depression, and anxiety, and then address PICO.

**Answer:** The systematic reviews included reported different definitions and considered different scales for diagnosing depression, depressive symptoms and anxiety in the studied population. This information is available in the S3 Appendix. We have modified the text of method to insert this information. We appreciated the suggestion and we are available to make to make any changes that the reviewer deems necessary.

*“****Population:*** *children, adolescents or adults with both type 1 or type 2 diabetes mellitus and with depression, depressive symptoms, and/or anxiety. Distress was also considered if the study population had depression or anxiety. We considered the diverse ways for diagnosing these diseases (clinical diagnosis or by using of different scales) reported in systematic reviews”.*

3- There are some concerns in PICO. It is said "psychoeducational interventions (including information and guidance for diabetes and/or psychological self-management" as intervention group and "non-pharmacological interventions may include
usual care or other interventions" as Comparators. In your mention "information and guidance" are pharmacological or non-pharmacological interventions? Considering that they are an example of non-pharmacological interventions, why is chosen
"non-pharmacological interventions as comparison group"? 4- What are exclusion criteria?

**Answer:** We've modified the text “Eligibility criteria” to make the information clearer. Studies could compare different non-pharmacological interventions. Thus, we found “usual care” or another non-pharmacological intervention as comparator group.

*Eligibility criteria*

*Inclusion criteria*

*Interventions:*

*non-pharmacological interventions: i) psychological (psychodynamic psychotherapy, interpersonal psychotherapy, non-directive counseling or support, among others); ii) psychoeducational (collaborative care, among others); iii) health education; iv) lifestyle interventions, among others;*

*pharmacological intervention: drugs used in the treatment of depression and/or anxiety.*

*Comparators:*

*non-pharmacological interventions: usual care or other non-pharmacological interventions;*

*pharmacological interventions: active control or placebo.*

*Outcomes: effectiveness and safety outcomes described in “Measure outcomes”.*

*Type of study: systematic review of randomized clinical trials followed by meta-analysis. Systematic reviews with more than one study design were included, but the collected information was restricted for those outcomes reported by randomized clinical trials.*

*Exclusion criteria*

*Type of study: systematic review in which interventions consisted only of adherence to diabetes treatment (although interventions to improve the adherence to diabetes have effects on mood, they were not designed to treat depression or anxiety. Review that contained clinical trials included in other reviews with the most recent publication date.*

5- The data on world prevalence of diabetes is old which should be updated.

**Answer:** Dear reviewer, we have updated the information as requested in “introduction”.

6- One of the limitations of the study is heterogeneity in anxiety assessment scales.

**Answer:** Dear reviewer, we have inserted this information in discussion “Study limitations”.

7-In search strategy, there are similarity between #3 and #4 and also between #5 and #6, which was better to delete common words before beginning search.
**Answer:** In “S1 Appendix. “Search strategy” we insert the Mesh terms used in the search performed in MEDLINE and adapted for the other databases. We inserted the terms without repetition, however, the database MEDLINE the file that we make available in Appendix S1.

Dear reviewer, we appreciated the suggestion and are at your disposal for any adjustments that the reviewer deems necessary.
